# Salinity significantly affects methane oxidation and methanotrophic community in Inner Mongolia lake sediments

**DOI:** 10.3389/fmicb.2022.1067017

**Published:** 2023-01-06

**Authors:** Shaohua Zhang, Lei Yan, Jiahui Cao, Kexin Wang, Ying Luo, Haiyang Hu, Lixin Wang, Ruihong Yu, Baozhu Pan, Ke Yu, Ji Zhao, Zhihua Bao

**Affiliations:** ^1^Ministry of Education Key Laboratory of Ecology and Resource Use of the Mongolian Plateau & Inner Mongolia Key Laboratory of Grassland Ecology, School of Ecology and Environment, Inner Mongolia University, Hohhot, China; ^2^Inner Mongolia Key Laboratory of Environmental Pollution Control & Waste Resource Reuse, Inner Mongolia University, Hohhot, China; ^3^Institute of Water Resources and Hydro-electric Engineering, Xi’an University of Technology, Xi’an, Shaanxi, China; ^4^School of Environment and Energy, Shenzhen Graduate School, Peking University, Shenzhen, China

**Keywords:** methanotrophic community, CH_4_ oxidation potential, co-occurrence, salinity, Inner Mongolia

## Abstract

Methanotrophs oxidize methane (CH_4_) and greatly help in mitigating greenhouse effect. Increased temperatures due to global climate change can facilitate lake salinization, particularly in the regions with cold semiarid climate. However, the effects of salinity on the CH_4_ oxidation activity and diversity and composition of methanotrophic community in the sediment of natural lakes at a regional scale are still unclear. Therefore, we collected lake sediment samples from 13 sites in Mongolian Plateau; CH_4_ oxidation activities of methanotrophs were investigated, and the diversity and abundance of methanotrophs were analyzed using real-time quantitative polymerase chain reaction and high throughput sequencing approach. The results revealed that the diversity of methanotrophic community decreased with increasing salinity, and community structure of methanotrophs was clearly different between the hypersaline sediment samples (HRS; salinity > 0.69%) and hyposaline sediment samples (HOS; salinity < 0.69%). Types II and I methanotrophs were predominant in HRS and HOS, respectively. Salinity was significantly positively correlated with the relative abundance of *Methylosinus* and negatively correlated with that of *Methylococcus*. In addition, CH_4_ oxidation rate and *pmoA* gene abundance decreased with increasing salinity, and salinity directly and indirectly affected CH_4_ oxidation rate *via* regulating the community diversity. Moreover, high salinity decreased cooperative association among methanotrophs and number of key methanotrophic species (*Methylosinus* and *Methylococcus*, e.g). These results suggested that salinity is a major driver of CH_4_ oxidation in lake sediments and acts by regulating the diversity of methanotrophic community and accociation among the methanotrophic species.

## Introduction

Methane (CH_4_) is one of the major greenhouse gases, accounting for approximately 20% of total greenhouse effect; its greenhouse effect is 25–35 times that of carbon dioxide (CO_2_; Kirschke et al., 2013; IPCC, 2014). Global emissions of CH_4_ into the atmosphere are 500–600 Tg per year, and approximately 69% of CH_4_ emissions are by microorganisms (Conrad, 2009). Lakes are important sources of global CH_4_ emissions; lakes sediments account for approximately 6–16% of total natural CH_4_ emissions worldwide (Bastviken et al., 2004). In lake ecosystems, CH_4_ is produced from the anoxic layer of sediments or water column by archaeal methanogens using decomposed organic matter. Methanotrophs can use CH_4_ as the only carbon and energy source, consuming a large amount of CH_4_ produced by the biosphere (Hanson and Hanson, 1996). Methanotrophs in surface layer sediments in lakes can convert approximately 93% of CH_4_ produced in deeper sediments into CO_2_, greatly alleviating greenhouse effect caused by CH_4_ emissions from lakes (Frenzel et al., 1990). Therefore, methanotrophs play an important role in reducing CH_4_ emissions from lakes.

Salinization is a widespread and increasing threat to both inland and coastal wetland ecosystems (Herbert et al., 2015). Soda and salt lakes account for up to 80% of inland ecosystem in certain regions (Grant and Jones, 2000). Additionally, increased temperatures due to global climate change can accelerate the evaporation of water from wetlands, which can lead to shrinkage of wetland areas on a global scale. Particularly, cold or hot semiarid regions, as well as the Mediterranean climate zone, would be strongly affected (IPCC, 2007; Jeppesen et al., 2020). For example, when a wetland is in a long-term dry state due to the reduction of the static water head, the saline groundwater conducts and evaporates upward; this leads to rapid increase in salinization rate of wetlands (Herbert et al., 2015). Nutritional imbalance and osmotic stress caused by high salinity have direct or indirect adverse effects on microbial activity, CH_4_ oxidation, and CH_4_ emissions (Hanson and Hanson, 1996; Sherry et al., 2016; Zhao et al., 2019). For instance, Sherry et al. (2016) reported that the CH_4_ oxidation rate of sediments decreased with increased salinity by performing a cultivation experiment. Recently, Zhou et al. (2021) reported that the abundance of genes related to methanogenesis and CH_4_ emissions were higher in highly saline industrial ponds than in control wetlands, suggesting that high salinity increases the activity of methanogenic archaea.

The activity, diversity, and abundance of methanotrophic bacteria are affected by various environmental variables, e.g., CH_4_ and CO_2_ levels, pH, total organic carbon (TOC), temperature, and salinity (Dumestre and Guezennec, 1999; He et al., 2012; Deng et al., 2017; Osudar et al., 2017; Lew and Glińska-Lewczuk, 2018). In addition to their direct effects, these environmental variables may influence other variables and may indirectly affect the composition of methanotrophic community and CH_4_ oxidation rate in grassland soil (Kou et al., 2017). Increasing temperature due to global climate change can increase wetland salinity (Herbert et al., 2015). Several studies have reported the effect of salinity on methanotrophic community and CH_4_ oxidation rate in sediments or paddy soil under laboratory conditions (Sherry et al., 2016; Ho et al., 2018). For example, salinization may indirectly affect the activity and community composition of methanotrophs by increasing the availability of ammonium nitrogen (NH_4_^+^-N) in paddy soil (Ho et al., 2018). Khmelenina et al. (2000) reported that highly alkaline conditions (pH 8.15–9.4) led to a reduction in CH_4_ oxidation activity in the sediments of soda lakes of southern Transbaikal. However, the relevant data on methanotrophic community and other environmental factors such as salinity were unavailable (Khmelenina et al., 2000). In addition, cultivation experiment revealed that CH_4_ oxidation activity and methanotrophic community of sediment were affected by pH, salinity, or temperature (Sherry et al., 2016). Recently, Deng et al. (2017) reported that when the salinity is >1.5 g/L, certain type I methanotrophic bacteria such as *Methylomicrobium* become the absolute dominant species (exhibiting up to 100% abundance) in alkaline lake sediments on the Tibetan Plateau. However, this study did not determine the effect of salinity on CH_4_ oxidation rate. To date, salt-stress-resistant methanotrophic strains have been isolated from various environments such as soda lakes (Heyer et al., 2005; Sorokin and Kuenen, 2005), alkaline lakes (Sorokin et al., 2000; Eshinimaeva et al., 2002), and sea water (Bowman et al., 1993), and most of them are type I methanotrophs belonging to the class of *Gammaporoteobacteria*. Thus, most studies on the influence of salinity on CH_4_ oxidation activity and methanotrophic community focus on local or control experiments in laboratory. At a regional scale, the effects of salinity on the CH_4_ oxidation and diversity and composition of methanotrophic community in the sediment of natural lakes, along with environmental variables, are still unclear.

The lakes of Inner Mongolia are mainly located in the cold regions of the Mongolian Plateau with a total lake area of 6151.2 km^2^, corresponding to 14.7% of the total number of lakes and 7.6% of the total lake area in China (Ma et al., 2011). The Inner Mongolian Plateau has arid and cold semiarid climate with scarce precipitation. IPCC (2014) reported that the regions with arid and cold semiarid climate will be strongly affected by global climate change. Due to the untimely recharge of surface runoff, evaporation rate of lakes is much higher than recharge rate, which is gradually turning many lakes into saltwater lakes or salt lakes (Zheng et al., 2002). Previous studies have confirmed that methanotrophs in Inner Mongolia steppe soils and some wetlands play an important role in reducing greenhouse effect (Ma et al., 2016; Kou et al., 2017; Liu et al., 2020). However, it is unclear whether salinity is the main factor influencing the characteristics of methanotrophic communities, and the major drivers affecting CH_4_ oxidation rate in the sediments of lakes in cold semiarid climate are not reported to date. Therefore, we collected sediment samples from 13 lakes in Inner Mongolia and investigated the effects of various environmental factors including salinity on the diversity and composition of methanotrophic communities and identified the major environmental factors influencing the CH_4_ oxidation potential and accociations among methanotrophic species.

## Materials and methods

### Study site and climate data collection

A total of 39 sediment samples were collected from 13 lakes from Inner Mongolia in September 2019. The vegetation types of sampling regions were forest, steppe, and desert from east to west. The annual average temperature (AAT) is from −0.4 to 9.5°C, and the annual rainfall is 22.9–460 mm. The sampling site locations and basic climate information are given in Supplementary Figure S1 and Supplementary Table S1, respectively. All sediment samples were collected by random sampling method according to the respective lake area. Each lake sample plot was divided into 3 areas. After collecting at least 3 sediment samples from each area, the sediments form each area were completely mixed; one part was stored in 50 ml sterile centrifuge tubes with liquid nitrogen in an ultra-low temperature freezer (−80°) for subsequent molecular biology analysis. The other parts were kept on dry ice, and their physical and chemical properties were determined after air drying, grinding, and screening in the laboratory.

### Physicochemical analysis of sediments

Physicochemical analysis of the sediment samples was performed as described in previous studies (Zhang et al., 2021a). NH_4_^+^-N and nitrate nitrogen (NO_3_^−^-N) from sediments were extracted using 2 mol L^−1^ KCl solution for 2 h and filtered. Their contents were determined using AMS automatic discontinuous chemical analyzer (AMS smartchem140, Italy). TOC and total nitrogen (TN) were measured using elemental analyzer (Vario EL Cube, Elementar, Germany). Total phosphorus (TP) content was evaluated using an inductively coupled plasma emission spectrometer (ICP6000, Thermo Fisher Scientific, United States). Water content (WC) in the sediment samples was determined by drying the samples at 105°C. Soil and water were mixed in a ratio of 1:5 to determine the pH using a pH meter. The salinity of sediments was assessed using the residue drying method. Each analysis was performed using three replicates for each sample to ensure data accuracy.

### DNA extraction, *pmoA* amplification, and amplicon sequencing

Total genomic DNA from approximately 0.5 g of each sediment sample was extracted using FastDNA Spin Kit for Soil DNA Extraction (MP Biomedicals, Solon, OH) as per the manufacturers’ instructions. The DNA concentration was determined using NanoPhotometer P-class ultrafine photometric analyzer (NanoPhotometer, Implen GmbH, Germany), and DNA samples were stored at −20°C for molecular biology analysis. *pmoA* gene was amplified using the barcode primer pair A189F/mb661R (Costello and Lidstrom, 1999) and reagent kit (RR902A, Premix ExTaq™, Takara Bio Inc., Japan) and was used to detect methanotrophs. The PCR amplification program is given in Supplementary Table S2. *pmoA* from the sediments was sequenced using the Illumina MiSeq platform (Shanghai Majorbio Technology Corporation, China).

The original sequences were spliced using FLASH 1.2.11 software. Quality control was performed using QIIME 1.9.1 software.^1^ The nucleotide sequences of *pmoA* were converted to amino acid sequences using the FunGene Pipeline of the Ribosomal Database Project (Wang et al., 2013). The sequences encoding proteins that did not contain the *pmoA* protein sequence or contained termination codons were discarded. Mothur^2^ was used to calculate the operational taxonomic units (OTUs) and diversity indices (including shannon index, sobs index, etc.) at a similarity level of 91% amino acid identity (Heyer et al., 2002). Phylogenetic trees were constructed using MEGA 7.0 software (Kumar et al., 2016). High-quality high-throughput sequencing data for *pmoA* genes were submitted to GenBank,^3^ with the accession number SRR18332830.

### Determination of CH_4_ oxidation potential

CH_4_ oxidation rates of sediments were determined using an incubation experiment (Kou et al., 2017). A 100 ml serum bottle was sterilized and dried. To that, 10 g fresh sediment sample was added, and the bottle was tightly closed with a rubber stopper and aluminum cap. Further, 2 ml of air was removed from the bottle using a sterile syringe, and CH_4_ gas was injected into it. The final mixing ratio was 2% (Siljanen et al., 2011). For culturing, the serum bottle was incubated in a shaker at 25°C in dark. Finally, CH_4_ concentration was measured using hydrogen ion flame detection gas chromatography (GC-2014, Shimadzu, Japan). The CH_4_ oxidation rate was determined according to the slope of the linear regression equation between CH_4_ oxidation and culture time (Shrestha et al., 2012). The control group did not contain sediment sample, and it was used to determine any gas leakage. The experiment was performed in triplicates of each sample.

### Determination of abundance of *pmoA*

The *pmoA* gene primer pairs A189F/mb661R were used to determine the abundance of *pmoA* gene in lake sediments using a CFX Connect Optical Real-Time Detection System (Bio-Rad laboratories, Hercules, United States) and SYBR® Premix Ex Taq TMII (Liu et al., 2021). The amplification system (20 μl) contained 10 μl SYBR Premix EX Taq enzyme (Takara Biotech, Dalian, China), 500 nM primers A189F and mb661R (Costello and Lidstrom, 1999), 2 μl DNA template, and DNase/RNase-free deionized water to make up the volume to 20 μl. Three replicates were used for each sample. The negative control was set by adding deionized water instead of DNA template. The amplification procedure was 30 s at 95°C (initial denaturation), followed by 35 cycles of 30 s at 95°C (denaturation), 45 s at 53°C (annealing), and 45 s at 72°C (extension; Supplementary Table S2). The PCR product of the sample DNA was cut, recovered, and cloned. The positive clone was selected and sequenced. Meanwhile, the plasmid was extracted after confirming the target gene sequence. The standard plasmid sample was obtained according to the 10-fold dilution method, and a standard curve was plotted. The amplification efficiencies were 95–98%. R^2^ values ranged between 0.999 and 1. No signals were observed in negative controls.

### Network analysis

To investigate the potential accociations, stability, and key members of methanotrophic community, co-occurrence network analyses were conducted at the OTU level though the Molecular Ecological Network Analyses Pipeline (MENAP, http://ieg4.rccc.ou.edu/MENA/main.cgi). Using the default settings and recommended similarity thresholds, networks were constructed and visualized in Gephi 0.9.2 (Wei et al., 2020). The keystone taxa of potential were identified based on the within-module connectivity (Zi) and among-module connectivity (Pi) of each node in the correlation networks (Zhang et al., 2019; Wei et al., 2020).

### Statistical analysis

The correlations among the Shannon index, Sobs index, CH_4_ oxidation rate, methanotrophic species, and environmental variables were determined using univariate models in SPSS software (version 20.0) according to Akaike information criteria (AIC) value (Fierer and Jackson, 2006). The one-way analysis of variance was performed using pair-wise least significant difference method in SPSS software (version 20.0, using a threshold of *p* < 0.05) under the criteria that the data met the test of homogeneity of variance (*p* > 0.05). To evaluate the dissimilarity in methanotrophic community between samples, nonmetric multidimensional scaling (NMDS) and UniFrac tree cluster analysis were conducted based on Bray–Curtis distance and Pairwise weighted UniFrac distance, respectively, with the “Vegan” package of R Studio. Pearson’s correlation analysis between methanotrophic community and environmental variables was conducted based on Bray–Curtis with the R Studio “Vegan” package (Wei et al., 2020). Redundancy analysis (RDA) and variance partitioning analysis (VPA) were performed to analyze the relationships between the environmental variables and methanotrophic community (based on OTU level) using CANOCO software (version 5.0). Structural equation models (SEM) were used to identify the relative importance and effects of the abundance of *pmoA*, environmental variables, and richness and diversity of methanotrophic community on CH_4_ oxidation using Amos software (version 26.0; Lange et al., 2015). The fitness of the final model was assessed using the chi-square/degrees of freedom (x^2^/df, <3), root mean square error of approximation (RMSEA, <0.05), and its associated *p* value (*p* > 0.05). To obtain a better fitted model, we calculated the comparative fit index (>0.95), Tucker–Lewis coefficient index (>0.90), and goodness of fit index (GFI, >0.90) and chose the final model that exhibited the lowest AIC.

## Results

### Evaluation of environment variables in the lake sediments

The environmental variables in the sediments were evaluated (Supplementary Figure S2). Salinity ranged from 0.53 to 43.12%; particularly, Badan (salt water) lake (BDS) sediments were hypersaline sediments. TN and TOC were 0.21–11.28 and 0.01–0.90 g/kg, respectively. The maximum value of TOC/TN was in Xilinhe (XLH; 3.95). TP was the highest in Hulun lake (HLH; 0.7 g/kg) and lowest in XLH (0.14 g/kg); however, no significant difference was observed in terms of TP among the samples. NH_4_^+^-N (mg/kg) in Wusulangzi lake (WSLZ) was significantly higher than that in other samples. NO_3_^−^-N (mg/kg) was the highest in Tonggunaoer Lake (TGNE). The WC of the sediments was 10–67%. The pH of the sediments was alkaline (pH > 7.0); particularly, Hongjiannao Lake (HJN; pH = 9.24) and TGNE (pH = 9.94) were highly alkaline.

### Diversity of methanotrophic community

A total of 776,859 sequences were obtained from 39 sediment samples by *pmoA* gene amplicon sequencing. Overall, 598,292 high-quality sequences were grouped into 110 representative OTUs. The diversity of methanotrophic community is summarized in Supplementary Table S3. The univariate models revealed that salinity was the best predictor of diversity (R^2^ = 0.41, *p* < 0.001; Table 1 and Supplementary Figure S3A) and richness (R^2^ = 0.21, *p* = 0.016; Supplementary Figure S3B) of methanotrophic community. In addition, the Pearson’s correlation analysis indicated that salinity was significantly negatively correlated with the diversity and richness of methanotrophic community (Supplementary Figure S4).

**Table 1:** Univariate regression models predicting the diversity of methanotrophic community.

Environment variables	Model type	R^2^	*p* value	AIC value	a	b	c
Salinity (%)	Quadratic	0.41	<0.001	−41.06	2.03	−0.11	0.00
TN (g/kg)	Quadratic	0.13	0.08	−30.07	1.64	−0.23	0.02
TOC (g/kg)	Quadratic	0.13	0.09	−30.00	1.24	−0.84	1.58
TOC/TN	linear	0.14	0.02	−30.53	0.15	1.20	−
TP (g/kg)	Quadratic	0.28	0.01	−34.73	2.6	−6.51	6.15
NH_4_^+^-N (mg/kg)	Quadratic	0.03	0.64	−25.64	1.31	−0.01	0.00
NO_3_^−^-N (mg/kg)	linear	0.08	0.09	−27.80	0.03	1.15	−
AAT (°C)	Quadratic	0.17	0.04	−65.86	2.23	0.04	−0.02
pH	Quadratic	0.00	0.97	−24.77	3.64	−0.52	0.03
WC (%)	Quadratic	0.19	0.02	−32.89	2.36	−5.59	5.52

The Shannon index is a quantitative indicator that describes species richness and evenness. Therefore, the diversity of methanotrophic community was estimated based on the Shannon index. Univariate regression analysis included linear (y = a + bx) and quadratic (y = a + bx + cx^2^) regression model. Akaike information criteria (AIC) value was calculated, and model with the minimum AIC value was selected. The minimum AIC values implied high reliability of this model (Fierer and Jackson, 2006). TN: total nitrogen; TOC: total carbon; TOC/TN: carbon to nitrogen ratio; TP: total phosphorus; NH_4_^+^-N: ammonium nitrogen; NO_3_^−^-N: nitrate nitrogen; AAT: annual average temperature; WC: water content.

### Composition of methanotrophic community

**Figure 1:**
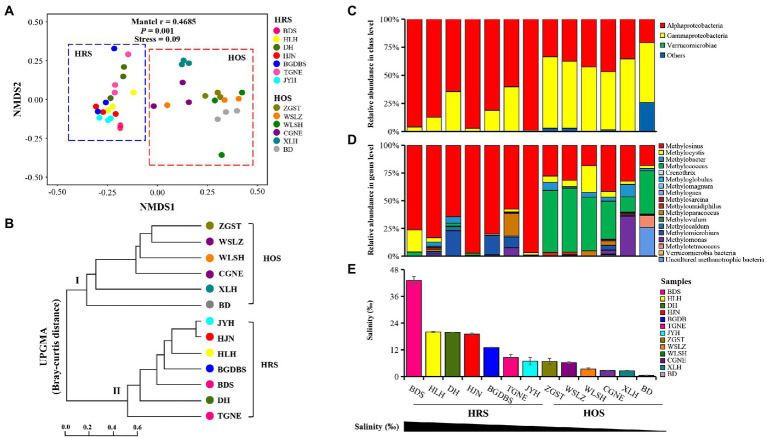
Nonmetric multidimensional scaling (NMDS) plots **(A)** and UniFrac tree (UPGMA; **B**) based on the Bray–Curtis distance in all sampling sites at the OTU level. Analysis of similarities was performed to evaluate the differences in the methanotrophic communities among the sampling sites. The community composition of methanotrophs at the class **(C)** and genus **(D)** levels in terms of salinity gradient **(E)**.

The results of NMDS and UniFrac tree cluster analysis revealed that the methanotrophic communities could be clearly distinguished into two groups: HRS (salinity: 0.69–43.12%) and HOS (salinity: 0.53–0.69%; Figures 1A,B), indicating that salinity exhibits significant effects on the methanotrophic community across all sites. Moreover, Mantel tests revealed a strong correlation between the methanotrophic community and salinity (mantel r = 0.4685; *p* = 0.001). With the change of the salinity gradient, the HRS and HOS exhibited clear differences.

Further analyses of methanotrophic phylogenetic composition revealed clear differences between HRS and HOS along the salinity gradient (Figures 1C,D). At the class level (Figure 1C), *Alphaproteobacteria* (relative abundance 64.46–98.90%) and *Gammaproteobacteria* (relative abundance 51.79–64.53%) were the dominant classes in HRS and HOS, respectively (Supplementary Table S4). At the genus level (Figure 1D), a type-II methanotroph *Methylosinus* (relative abundance 57.56–97.06%) was dominant in HRS, and the dominant species (OTU486) similarity to *Methylosinus* sp. D28 (Supplementary Figure S6). In contrast, *Methylococcus* and *Methylosinus* (relative abundance 13.79–57.52% and 18.31–41.64%, respectively) were mainly abundant in HOS (Supplementary Table S4).

**Figure 2:**
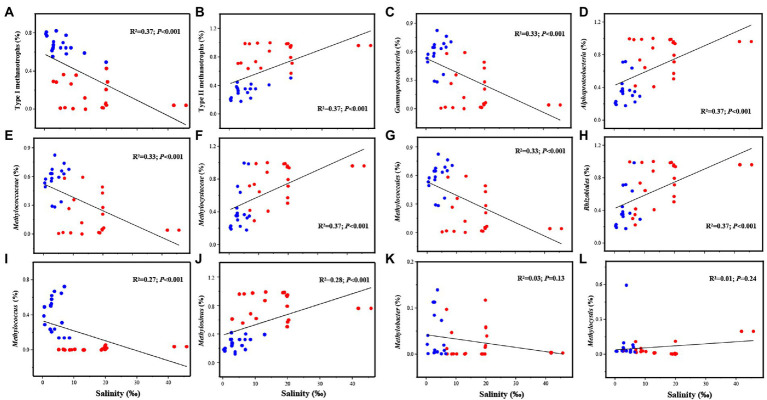
Linear regression analysis of the relationship between salinity and the dominant methanotrophs (with relative abundance >1%), including Type I methanotrophs **(A)**, Type II methanotrophs **(B)**, *Gammaproteobacteria* **(C)**, *Alphaproteobacteria* **(D)**, *Methylococcaceae* **(E)**, *Methylocystaceae* **(F)**, *Methylococcales* **(G)**, *Rhizobiales* **(H)**, *Methylococcus* **(I)**, *Methylosinus* **(J)**, *Methylobacter* **(K)**, *Methylocystis* **(L)**.

The results of the linear regression analysis suggested that the relative abundance of methanotrophs significantly correlated with salinity at the class, order, family, and genus levels (Figure 2). The relative abundance of type I methanotrophs exhibited a decreasing trend and that of type II methanotrophs exhibited an increasing trend with increasing salinity (Figures 2A,B). The abundance of *Gammaproteobacteria*, *Methylococcaceae*, and *Methylococcales* decreased and that of *Alphaproteobacteria*, *Methylocystaceae*, and *Rhizobiales* increased as salinity increased (Figures 2C–J). At the genus level, as salinity increased, *Methylosinus* exhibited an increasing and *Methylococcus* exhibited a decreasing trend; however, *Methylobacter* and *Methylocystis* were not affected by salinity (Figures 2K, L). Similar results were obtained in Pearson’s correlation analysis (Supplementary Figure S4). Moreover, salinity was significantly positively correlated with type II methanotrophs including *Methylosinus* (*p* < 0.01) and significantly negatively correlated with type I methanotrophs including *Methylococcus* (*p* < 0.01; Supplementary Table S5).

### Effect of environmental variables on CH_4_ oxidation rate

**Figure 3:**
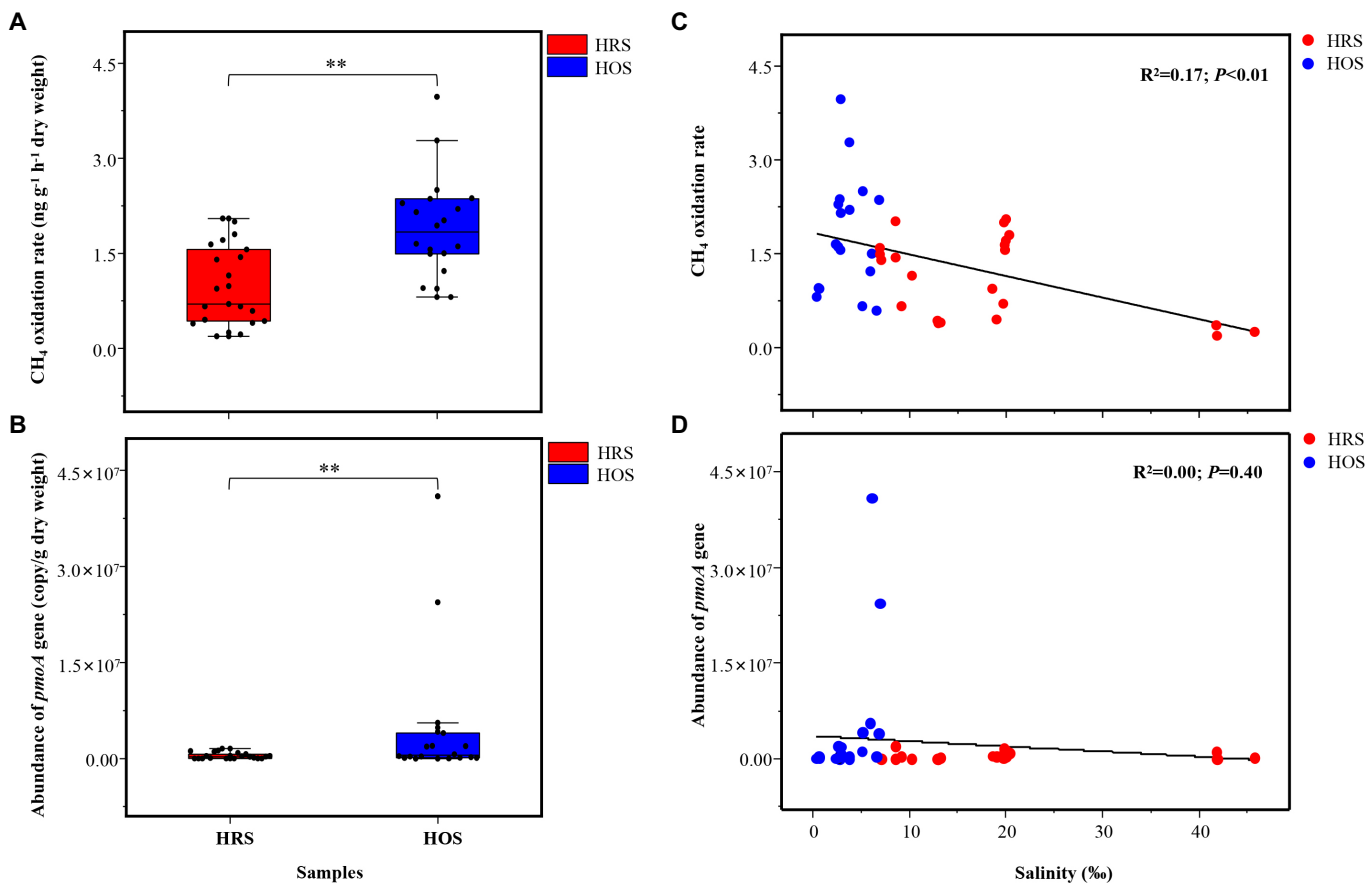
CH_4_ oxidation rate **(A)** and abundance (*pmoA* gene; **B**) of methanotrophs in HRS and HOS samples. The full site names of the sampling points are listed in Supplementary Table S1. The relationship among salinity, CH_4_ oxidation rate **(C)**, and abundance of *pmoA* gene **(D)**. Linear regression (y = a + bx) model was selected. The Akaike information criteria (AIC) value was calculated, and the model with the lowest AIC value was selected. The minimum AIC values implied high reliability of this model (Fierer and Jackson, 2006). Significant correlation: **p < 0.01 and *p < 0.05.

CH_4_ oxidation rate ranged from 0.22 ± 0.02 to 3.15 ± 0.73 ng g^−1^ dry weight h^−1^ in all lake sediments, and it was significantly different between HRS and HOS (*p* < 0.01; Figure 3A). The linear regression analysis (R^2^ = 0.17, *p* < 0.01; Figure 3C) and Pearson’s correlation analysis (*p* < 0.01; Supplementary Figure S4) revealed that salinity negatively affected CH_4_ oxidation rate. TN (*p* < 0.05), TOC (*p* < 0.05), and WC (*p* < 0.05) were positively correlated with CH_4_ oxidation rate (Supplementary Figure S4).

### *pmoA* gene abundance in methanotrophs

The *pmoA* gene copy numbers were 1.13 × 10^4^ to 2.38 × 10^7^ copies g^−1^ dry weight in all sediment samples, and they were significantly different between HRS and HOS (*p* < 0.01; Figure 3B). However, linear regression analysis indicated no significant relationship between salinity and *pmoA* gene abundance (R^2^ = 0.00, *p* = 0.40; Figure 3D). Pearson’s correlation analysis revealed that TOC (*p* < 0.01), TN (*p* < 0.01), NH_4_^+^-N (*p* < 0.01), and AAT (*p* < 0.01) were significantly positively correlated with *pmoA* gene abundance (Supplementary Figure S4).

### Environmental factors affecting the diversity, richness, and CH_4_ oxidation rate of methanotrophic community

RDA revealed that RDA1 and RDA2 corresponded to 40.71 and 8.43% of the total variation in the methanotrophic community structures, respectively (Supplementary Figure S7). The results demonstrated that the distribution of methanotrophs in the lake sediments was mainly driven by salinity, AAT, TN, and TOC/TN (Table 2). The VPA results indicated that the evaluated environmental variables accounted for 59.1% of the changes in methanotrophic community (Table 2). Among all the environmental factors, salinity was the most essential environmental factor affecting the community of methanotrophs, with an explanation degree of 23.3%. followed by AAT, TN, and TOC/TN (explanation degrees of 12.8, 7.1, and 7.0%, respectively).

**Table 2:** Contribution of environmental factors to methanotrophic community structure.

Environmental factors	Contribution (%)	*p* values
Salinity (%)	23.3**	0.001
AAT (°C)	12.8**	0.002
TN (g/kg)	7.1**	0.004
TOC/TN	7.0**	0.004
TP (g/kg)	3.5	0.086
pH	2.6	0.132
TOC (g/kg)	1.2	0.402
WC (%)	0.8	0.602
NH_4_^+^-N (mg/kg)	0.5	0.800
NO_3_^−^-N (mg/kg)	0.2	0.976
Combined effect of all factors	53.1	-

VPA analysis was performed to analyze the relationships between the environmental variables and methanotrophic community (based on OTU level). Montecarlo test with 999 permutations was used to determine significant difference. TN: total nitrogen; TOC: total carbon; TOC/TN: carbon to nitrogen ratio; TP: total phosphorus; NH_4_^+^-N: ammonium nitrogen; NO_3_^−^-N: nitrate nitrogen; AAT: annual average temperature; WC: water content. Significant correlation: **p < 0.01 and *p < 0.05; non-significant correlation: *p* > 0.05.

**Figure 4:**
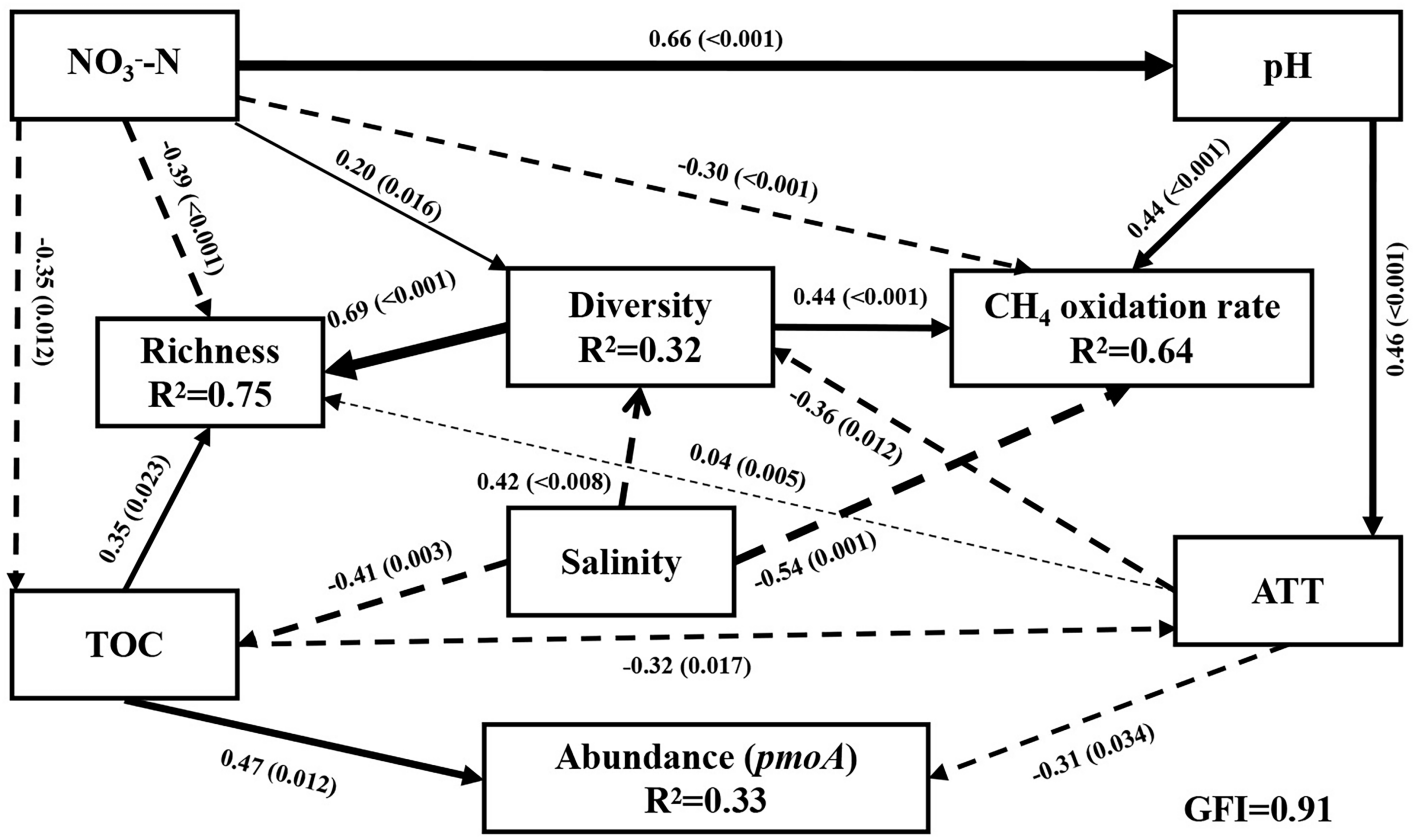
Structural equation models (SEM) between community diversity, richness, abundance, and CH_4_ oxidation rate of methanotrophs and environmental variables (x^2^/df = 0.97, *p* = 0.481, RMSEA = 0.00, GFI = 0.91, TLI = 1.00, CFI = 1.00, AIC = 71.62). Lower AIC values implied a better model fit. Solid black arrows and dashed black arrows represent positive and negative paths, respectively. R^2^ values associated with response variables indicate the proportion of variation explained by relationships with other variables. Values associated with arrows represent standardized path coefficients. *p*-values are indicated in parentheses. TOC: total carbon; TOC/TN: carbon nitrogen ratio; NO_3_^−^-N: nitrate nitrogen; Diversity: the Shannon index; Richness: Sobs index.

The environmental factors influencing the methanotrophic community diversity, richness, abundance, and CH_4_ oxidation rate were further determined by SEM. The modeling analysis provided the best fit to our data (Figure 4; GFI = 0.91). The results revealed that 32% variation in community diversity was accounted by salinity (Figure 4), NO_3_^−^-N, and AAT. The total effect of salinity (path coefficient = 0.54) on community diversity was greater than that of NO_3_^−^-N and AAT (path coefficient = 0.20 and 0.36, respectively). Overall, 75% variation in richness was accounted by NO_3_^−^-N, TOC, and AAT (path coefficient = 0.40, 0.31, and 0.04, respectively). Salinity indirectly affected the richness by affecting the TOC and diversity. Overall, 64% variation in CH_4_ oxidation rate was accounted by salinity, pH, and NO_3_^−^-N (path coefficient = 0.54, 0.44, and 0.30, respectively; Figure 4). CH_4_ oxidation rate was significantly negatively correlated with salinity and NO_3_^−^-N (*p* < 0.01) and significantly positively correlated with pH (*p* < 0.01). However, the results of Pearson’s correlation analysis revealed no significant correlation of pH and NO_3_^−^-N with CH_4_ oxidation rate. Different analysis methods may lead to different results. In addition, ATT indirectly affected the CH_4_ oxidation rate by affecting the methanotrophic community diversity. TOC had the highest effect on *pmoA* gene abundance (path coefficient = 0.47), and salinity indirectly affected *pmoA* gene abundance by affecting the TOC. Moreover, community diversity had a direct effect on CH_4_ oxidation rate and richness, and diversity was significantly positively correlated with CH_4_ oxidation rate and abundance (*p* < 0.001). Combining all the observations, the SEM results indicated that salinity was the most important environmental variable responsible for the variation in methanotrophic diversity and CH_4_ oxidation rate. The effect of salinity on the richness and abundance of methanotrophic community was secondary to that of NO_3_^−^-N and TOC.

### The key species of methanotrophs and correlation network structure

**Figure 5:**
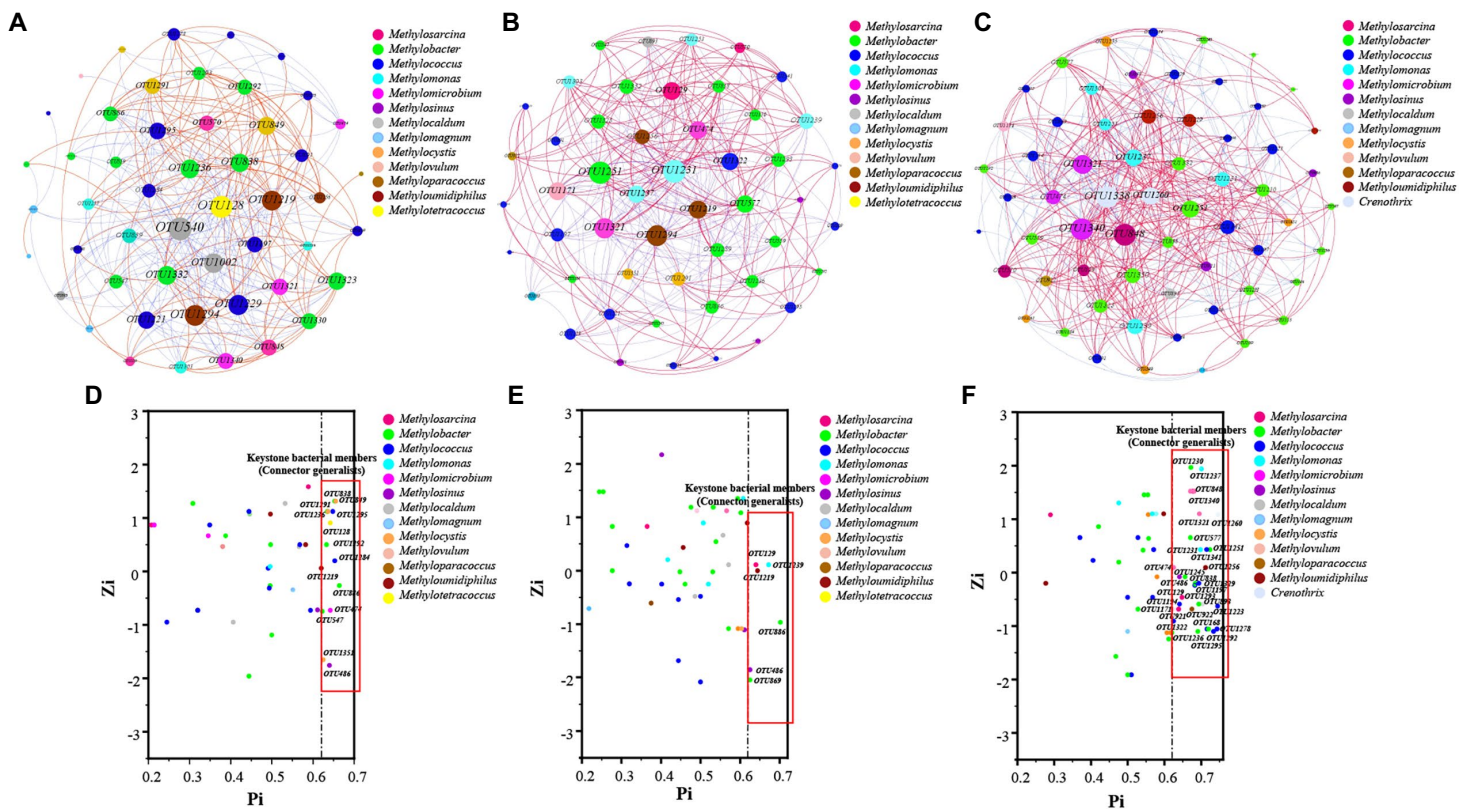
The co-occurrence network accociations of methanotrophic community based on OTU level of all samples **(A)**, hypersaline sediment samples (HRS; **B**), and hyposaline sediment samples (HOS; **C**). Red and blue indicate positive and negative correlations, respectively. Zi (within-module connectivity) and Pi (among-module connectivity) plots uncovering the key species of methanotrophic community of all samples **(D)**, HRS **(E)**, and HOS **(F)**.

The network property results revealed that negative correlations (58.84%) were higher than positive correlations (41.16%; Figure 5A). However, the positive edges proportions of HOS were higher than those of HRS (Figures 5B,C; Supplementary Table S6). Similarly, the node connectivity (average degree of 14.516), networks diameters (1.687), and average path length (1.93) were the highest in HOS compared with HRS. This suggested that the node degree centrality and closeness centrality of HOS network were higher than those of the HRS network.

The Zi quantifies the degree of connectivity between the nodes. The Pi quantifies the extent to which the nodes are connected to various modules (Zhang et al., 2019). As shown in Figures 5D–F, most of the nodes belonged to peripherals specialists (Zi ≤ 2.5; Pi ≤0.62), module hubs (Zi > 2.5; Pi ≤0.62), and network hubs (Zi > 2.5; Pi >0.62). A few nodes were connector generalists (Zi ≤ 2.5; Pi >0.62). The connector organizes various modules into a complete community and is considered a key member of the network (Zhang et al., 2019, 2021b). In this study, the connectors included 14 (in all samples), 6 (in HRS), and 33 (in HOS) OTUs; the node number of connectors generalists in HRS were significantly fewer than that in HOS (Supplementary Table S7). In HRS, *Methylosinus* (OTU486; relatively abundance 80.2%), *Methylosarcina* (OTU129), *Methylobacter* (OTU886, OTU869), *Methylomonas* (OTU1239), and *Methyloumidiphilus* (OTU1219) were the key members (Supplementary Table S7). In HOS (Supplementary Table S7), the key members were mainly *Methylobacter* (OTU577, OTU838, OTU1230, OTU1236, OTU1245, OTU1251, OTU1292, and OTU1293), *Methylococcus* (OTU1223; relatively abundance 20.0%), OTU1295, OTU1322, OTU1197, OTU1329, OTU1341, OTU1194, and OTU1278), and *Methylosinus* (OTU486: relatively abundance 28.1%, OTU837, and OTU921). Moreover, the key members contained some species with relatively low abundance such as *Methylosarcina*, *Methylomicrobium*, *Methylomonas*, *Crenothrix*, *Methyloparacoccus*, *Methyloumidiphilus*, *Methylovulum*, and *Methylocaldum*. Thus, high salinity reduced the number of key methanotrophic species.

## Discussion

Methanotrophs play an important role in reducing CH_4_ emission from wetlands into atmosphere. However, the key factors controlling the diversity, composition, and CH_4_ oxidation rate of methanotrophic community in the lake sediments across Inner Mongolia in Mongolian Plateau are unclear. In this study, we reported that salinity is the major factor controlling the CH_4_ oxidation rate by affecting methanotrophic community.

### Salinity affects methanotrophic community structure

Previous studies reported that a type-I methanotroph *Methylomicrobium* is the most dominant in salt lakes (Deng et al., 2017), and *Methylobacter* (type I methanotroph) and *Methylocystis* (type II methanotroph) are the predominant methanotrophs in freshwater lakes (Lin et al., 2004; He et al., 2012). Annual average temperature of Inner Mongolia is −0.4 to 9.5°C. The temperature in cold areas does not seem to be conducive to type II methanotrophs (optimum temperature for growth >15°C) but to type I methanotrophs (optimum temperature for growth = 0–10°C; He et al., 2012). However, our results revealed that when salinity was >0.69%, *Methylosinus* (type-II methanotroph) was the absolute dominant taxa. This salinity level appears to be the threshold, with the highest relative abundance of *Methylococcus* (type-I methanotroph) and *Methylosinus* (type-II methanotroph) in samples with salinity <0.69% (Figures 1C,D). Previous studies have confirmed that *Methylococcus* is widely present in wetland ecosystems (Yun et al., 2012, 2013). However, *Methylococcus* was not detected in alkaline hypersaline lakes (Lin et al., 2004; Deng et al., 2017). So far, all known species of *Methylococcus* can grow under 1% salinity conditions (Bowman et al., 1993). Most of the OTUs related to *Methylococccus* were classified as uncultured species, and they were mainly distributed in HOS in this study (salinity <0.69%, Supplementary Figure S5). Higher salinity (>0.69%) is more conducive to the growth of *Methylosinus*, and lower salinity is more conducive to the mutual accociations and growth of type II (*Methylosinus* and *Methylocystis*) and type I (*Methylococcus*, *Methylobacter*, and *Methylomonas*, etc.) methanotrophs. In addition, phylogenetic analysis revealed that major OTUs were close to *Methylosinus trichosporium* OB3b (Supplementary Figure S6). *Methylosinus trichosporium* OB3b could grow at <2% NaCl (Bowman et al., 1993), which was consistent with our result. In addition, Han et al. (2017) reported that *Methylocystis* sp. strain SC2 (type-II methantoroph) could cope with salt stress at <1% NaCl, and this salt stress induced expression of differentially expressed genes in this strain SC2. The products of the transcriptional stress response major controller (e.g., sigma factor σ^32^) can control the stress response or confer stress tolerance under salt stress (Han et al., 2017). Specific cellular and biochemical properties of methanotrophs are altered under long-term salt stress; these properties include the synthesis of osmoprotectants, potassium ions accumulation, formation of glycoprotein S-layers on the outer surface of their cell walls, and modification of the chemical composition of their membranes. These alterations enable them to adapt to highly saline environments (Khmelenina et al., 1999; Trotsenko and Khmelenina, 2002). Methanotrophic abundance in HOS was significantly higher than that in HRS, which suggested that long-term salt stress was not conducive to poorly salt-tolerant methanotrophs. Similar results were obtained in the sediments of Qinghai lake (Deng et al., 2017). Therefore, we speculated that *Methylosinus* may have a special mechanism to overcome long-term salt stress to become the absolutely dominant taxa. Types I and II halophilic and salt-tolerant methanotrophs were gradually isolated form soda lake (Khmelenina et al., 1997; Carini et al., 2005; Eshinimaev et al., 2008), and pure-culture study was performed to assess resilience of methanotrophs to salt stress (Han et al., 2017; Ho et al., 2018). However, so far, these studies have focused on single laboratory conditions (e. g., pH and NaCl). The coping strategies of methanotrophs to salt stress under the influence of multiple environmental factors need to be studied.

### Salinity affects CH_4_ oxidation rate and *pmoA* gene abundance in methanotrophs

Although our results demonstrated that salinity has no effect on the abundance of *pmoA* (Figure 3D), a previous study reported that salinity exhibited negative effect on *pmoA* gene abundance in saline lakes (Deng et al., 2017). We determined the CH_4_ oxidation rate of the sediment, which was significantly negatively affected by salinity (Figure 3C). Yang et al. (2019) reported that CH_4_ oxidation rate of the sediment with high temperature (summer season) was higher than that of the sediment with lower temperature (winter and spring seasons). However, *pmoA* gene abundance in summer was lower than that in winter and spring. The different responses of *pmoA* gene abundance and CH_4_ oxidation rate to salinity or temperature may be related to gene abundance at DNA level. Determining RNA level is often better than determining DNA level because it is targeted at functional bacteria such as methanotrophs or diazotrophs with physiological activity (Cai et al., 2016; Cui et al., 2020, 2022). Therefore, compared with the *pmoA* gene abundance at the DNA level, the transcription level or CH_4_ oxidation rate can better reflect of the methanotrophic community activity.

CH_4_ oxidation rate decreased with increasing salinity (Figure 3). The same trend was exhibited at 21 and 40°C in estuarine sediments (Sherry et al., 2016). Besides, the changes in CH_4_ oxidation rate may be related to the composition of methanotrophic community in soil (Lin et al., 2005; Kou et al., 2017). The increase in salinity resulted in a reduced methanotrophic community diversity (Supplementary Figure S3). Various methanotrophic taxa exhibit different levels of tolerance to salinity (Bowman et al., 1993; Ho et al., 2018). Thus, CH_4_ oxidation rate may exhibit different trends under different salinity conditions, e.g., *Methylosinus* was resistant to the increase in salinity in lake sediments with high salinity (Lin et al., 2004; Osudar et al., 2017). In addition, *Methylosinus* grew well with salinity of <0.3 M NaCl (1.75% salinity). In case of >0.6 M NaCl (3.5% salinity), the CH_4_ oxidation rate of *Methylosinus* decreased significantly and was even completely abolished (Osudar et al., 2017; Ho et al., 2018). In addition, growth and CH_4_ oxidation rate of *Methylocystis* were clearly inhibited as salinity increased (0.5–1% NaCl; Han et al., 2017). This can explain why the CH_4_ oxidation rate decreased as salinity increased, although type II methanotrophs, particularly *Methylosinus*, were dominant in HSR (Figure 1D; Supplementary Table S4).

SEM analysis (Figure 4) revealed that salinity had a stronger effect on CH_4_ oxidation rate than NO_3_^−^-N, ATT and pH. Besides, we observed that salinity had an indirect effect on CH_4_ oxidation rate *via* affecting methanotrophic community diversity. Moreover, other studies indicated that environmental factors (e.g., TN and TOC) may influence grassland soil CH_4_ oxidation rate through their effects on methanotrophic community structure (Kou et al., 2017). CH_4_ oxidation rate increased in soil when the composition or abundance of methanotrophs increased (Steenbergh et al., 2009). Indeed, in our study, methanotrophic community diversity, abundance, and CH_4_ oxidation rate were significantly higher in HOS than in HRS. Salinity was significantly negatively correlated with methanotrophic diversity. A significant positive correlation existed between methanotrophic diversity and CH_4_ oxidation rate, which may explain the dual (direct and indirect) effects of salinity on CH_4_ oxidation rate. In addition, CH_4_ oxidation rate was significantly negatively correlated with NO_3_^−^-N. This result was consistent with the previous research showing that the increase of nitrogen concentration such as NO_3_^−^-N inhibited CH_4_ oxidation rate in eutrophication lake sediments (Yang et al., 2019). Moreover, ATT indirectly influenced CH_4_ oxidation rates by influencing diversity of methanotrophs community (Figure 4; Supplementary Figure S4). Some studies have reported that CH_4_ oxidation rates increase with methanotrophic community diversity (Yang et al., 2019; Bhardwaj et al., 2020) because increasing temperature (4–30°C) increased the methanotrophic community diversity in laboratory experiments (Sherry et al., 2016). However, the CH_4_ oxidation rates did not increase gradually with the ATT in our study. This may be the result of multiple effects of environmental factors, and the limitation of microbial activity by certain environmental factors may be alleviated by other environmental factors (Yue et al., 2019). Thus, determination of CH_4_ oxidation rate in natural environments at a regional scale is more complicated than that in a lake or cultivation experiment because of various environmental factors.

### High salinity decreased co-occurrence network relationship among methanotrophs

Positive and negative correlations of co-occurrence networks can be used to assess the collaboration or competitive relationships between bacterial species; the relationships among coexisting microorganisms reflect microbial responses to the environment (Weiss et al., 2016; Zhang et al., 2021b). The regulation of accociation between microorganisms under the condition of environmental pollution may be a survival strategy (Luo et al., 2022). In our study, the number of the positive correlations between methanotrophic taxa in HOS was greater than those in HRS (Figure 5; Supplementary Table S6), indicating that the network of methanotrophs was more closely connected under low-salinity conditions. Previous studies reported that increased salinity of lake waters leads to increased number of correlations among the bacterial taxa, based on 16S rRNA gene (Ji et al., 2019). This is inconsistent with our result (decreased correlations among methanotrophs under increased salinity condition in HRS). This could be because methanotrophic diversity is directly related to the accociations among methanotrophs (Zhang et al., 2019); HRS and HOS exhibited clear differences in terms of methanotrophic community diversity (Supplementary Figure S5). In addition, increased salinity reducing methanotrophic diversity may influence network correlations among methanotrophic taxa (Figure 1; Supplementary Figure S4). On the other hand, salinity has different effects on the network co-occurrence relationships among species of bacterial communities, based on 16S rRNA gene and functional gene such as *pmoA*. The differences in the distribution of methanotrophic communities would inevitably lead to changes in network relationships among methanotrophs. The accociation among bacteria enhances their tolerance to environmental changes; however, this tolerance does not completely overcome the influence of environmental factors (Ji et al., 2019) when an environmental factor reaches a threshold value. Salt tolerant bacteria and halophiles gradually become dominant under high salinity conditions, whereas bacteria with poor adaptability to high salinity gradually become dormant or exhibit reduced abundance (Aanderud et al., 2016). This view is confirmed by the reduced number of key species in HRS (Figures 5E,F; Supplementary Table S7). In general, high salinity reduces the mutual accociations between methanotrophs and reduces the number of key species. However, it should be further studied whether the reduction in key species will lead to enhanced CH_4_ emission because the CH_4_ oxidation potential of various methanotrophs under various salt stresses varies (Ho et al., 2018).

In conclusion, the results of this study revealed the key environmental factors affecting diversity, abundance, and CH_4_ oxidation rate of methanotrophs in lake sediments in Inner Mongolia. The methanotrophic community composition was clearly different in HRS and HOS, and types II and I methanotrophs were the clearly dominant taxa, respectively. As salinity increased, the abundance of *Methylosinus* (type II methanotroph) increased and that of *Methylococcus* (type I methanotroph) decreased. In addition, high salinity reduced the CH_4_ oxidation rate, accociations among methanotrophic species, and number of key members of methanotrophs. Salinity was the major environmental factor controlling CH_4_ oxidation rate, and it acted by regulating methanotrophic community structure and accociations among methanotrophic species. Therefore, increased salinity in lake sediments reduced CH_4_ oxidation and may influence one-carbon cycle, aggravating global warming. Future studies should analyze the effect of change in salinity on global carbon cycle, particularly on CH_4_ emission, in natural environment.

## Data availability statement

The datasets presented in this study can be found in online repositories. The names of the repository/repositories and accession number(s) can be found at: https://www.ncbi.nlm.nih.gov/genbank/, SRR18332830.

## Author contributions

ZB, LW, RY, BP, and KY designed the study. SZ and LY performed the experiments. SZ, JC, KW, YL, JZ, and ZB analyzed the data. SZ and ZB wrote the paper. All authors contributed to the article and approved the submitted version.

## Funding

This study was funded by the Science and Technology Major Project on Lakes of Inner Mongolia grant (No. ZDZX2018054), National Natural Science Foundation of China grants (No. 32160028, 32160279 and 32161143025), and Major Science and Technology Projects in Inner Mongolia Autonomous Region (No. 2022YFHH0086 and 2022YFHH0017).

## Conflict of interest

The authors declare that the research was conducted in the absence of any commercial or financial relationships that could be construed as a potential conflict of interest.

## Publisher’s note

All claims expressed in this article are solely those of the authors and do not necessarily represent those of their affiliated organizations, or those of the publisher, the editors and the reviewers. Any product that may be evaluated in this article, or claim that may be made by its manufacturer, is not guaranteed or endorsed by the publisher.

## Supplementary material

The Supplementary material for this article can be found online at: https://www.frontiersin.org/articles/10.3389/fmicb.2022.1067017/full#supplementary-material

## Footnotes

^1^ https://qiime2.org/

^2^ https://mothur.org/

^3^ http://www.ncbi.nlm.nih.gov/BankIt/
