# Supplementary figures and images for: Salinity significantly affects methane oxidation and methanotrophic community in Inner Mongolia lake sediments
Source: Front Microbiol. 2023 Jan 6;13:1067017. doi: 10.3389/fmicb.2022.1067017 (PMC9853545; doi:10.3389/fmicb.2022.1067017)

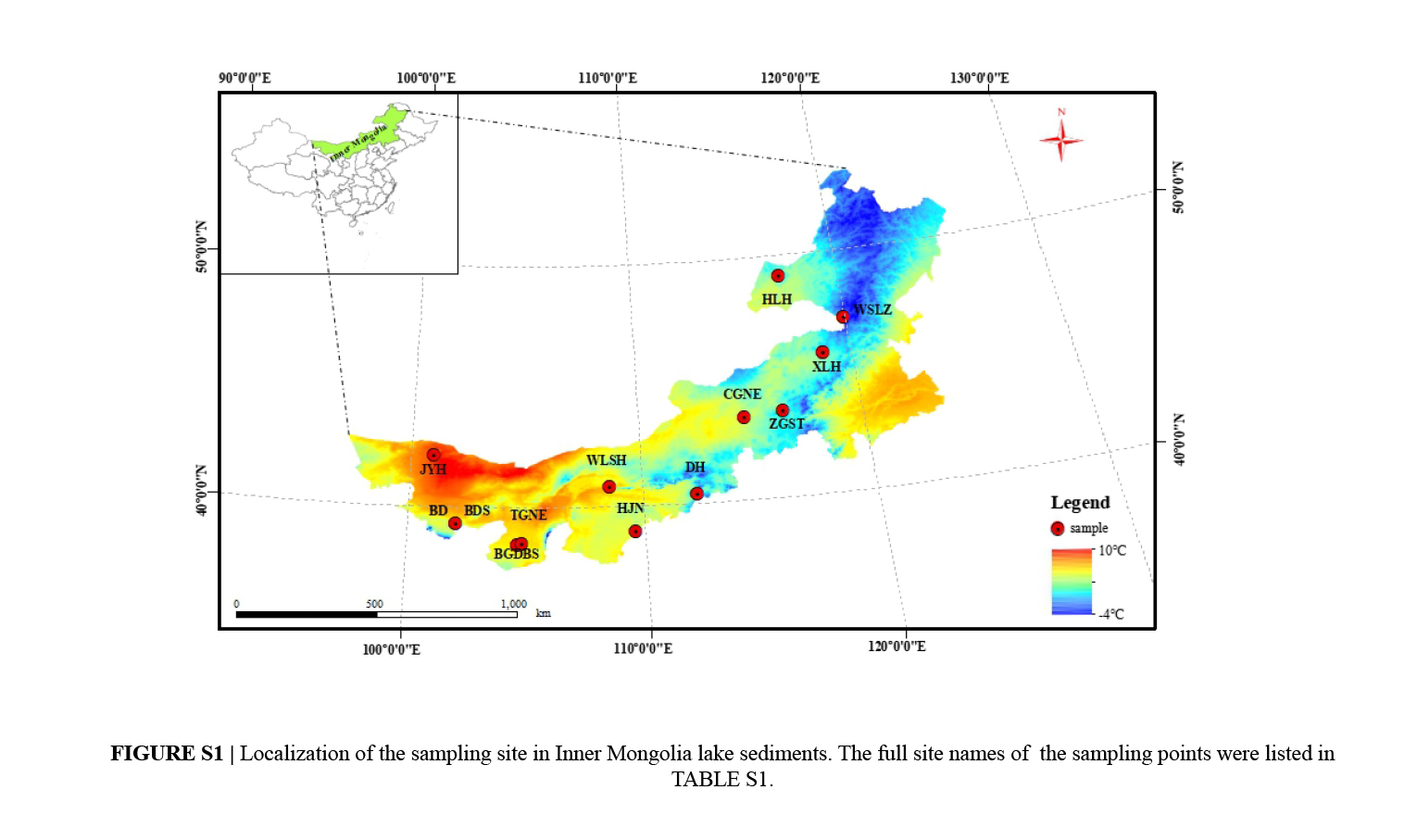

Supplement: Supplementary file 2 [file Image_1.JPEG]

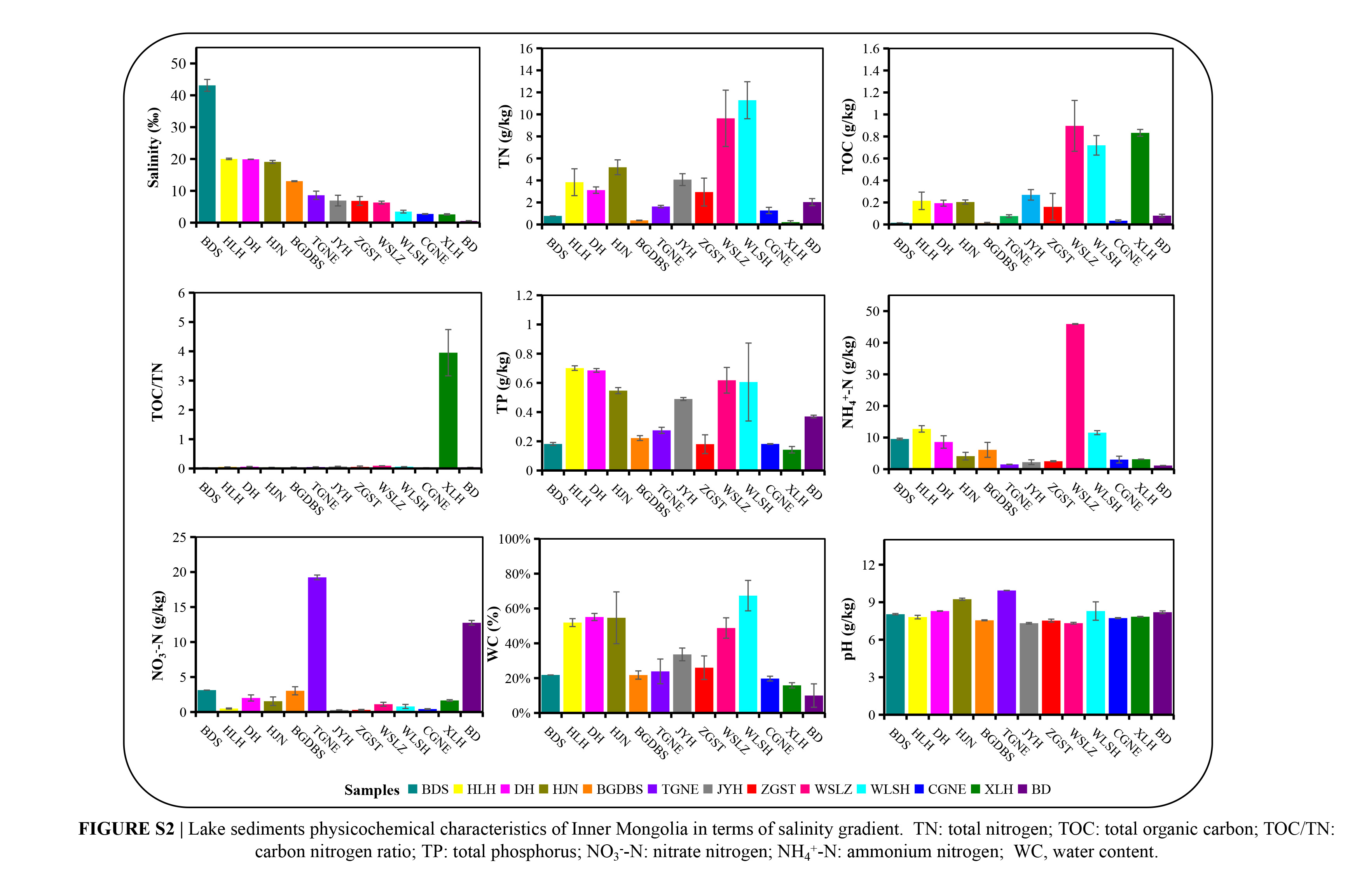

Supplement: Supplementary file 3 [file Image_2.JPEG]

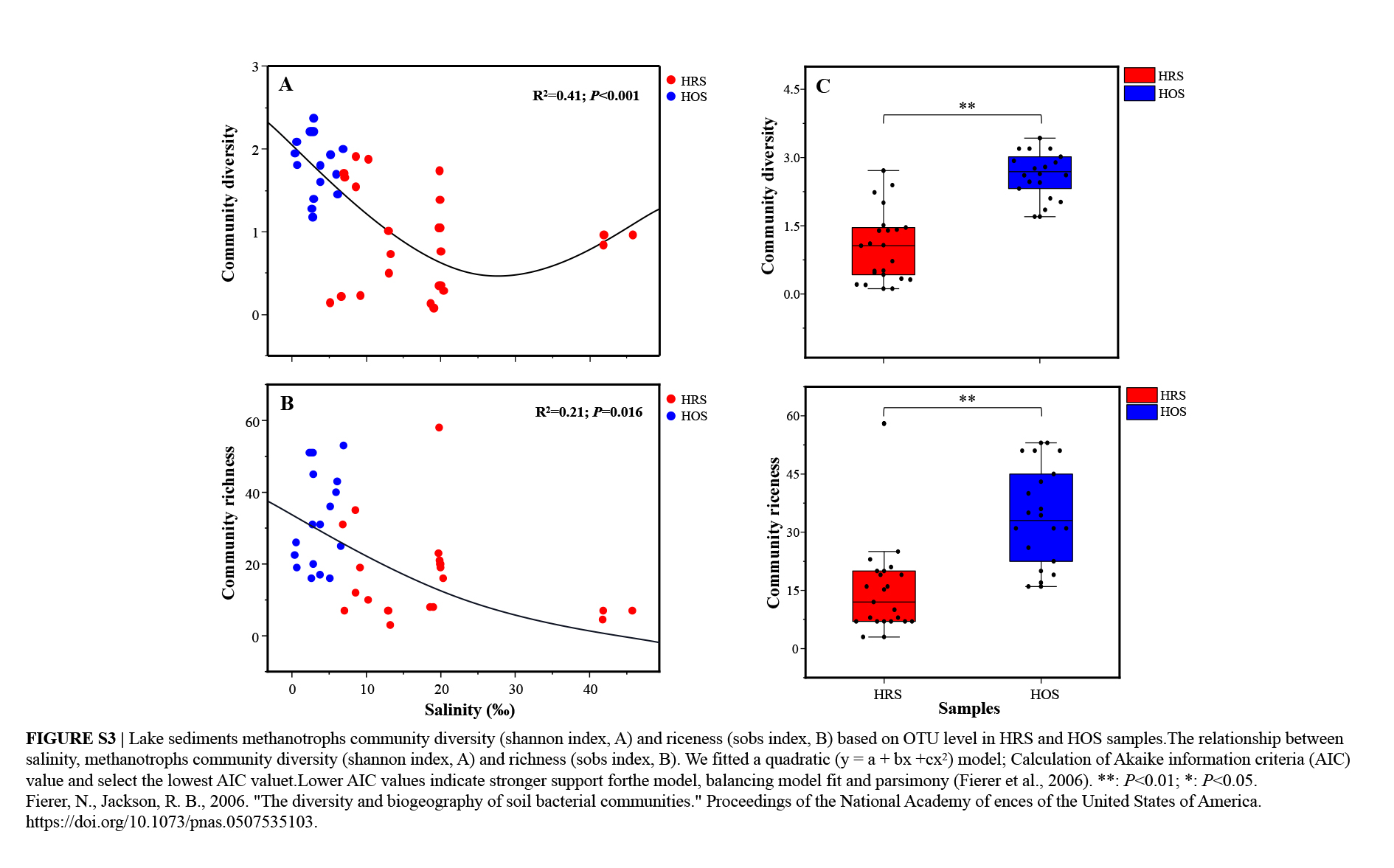

Supplement: Supplementary file 4 [file Image_3.JPEG]

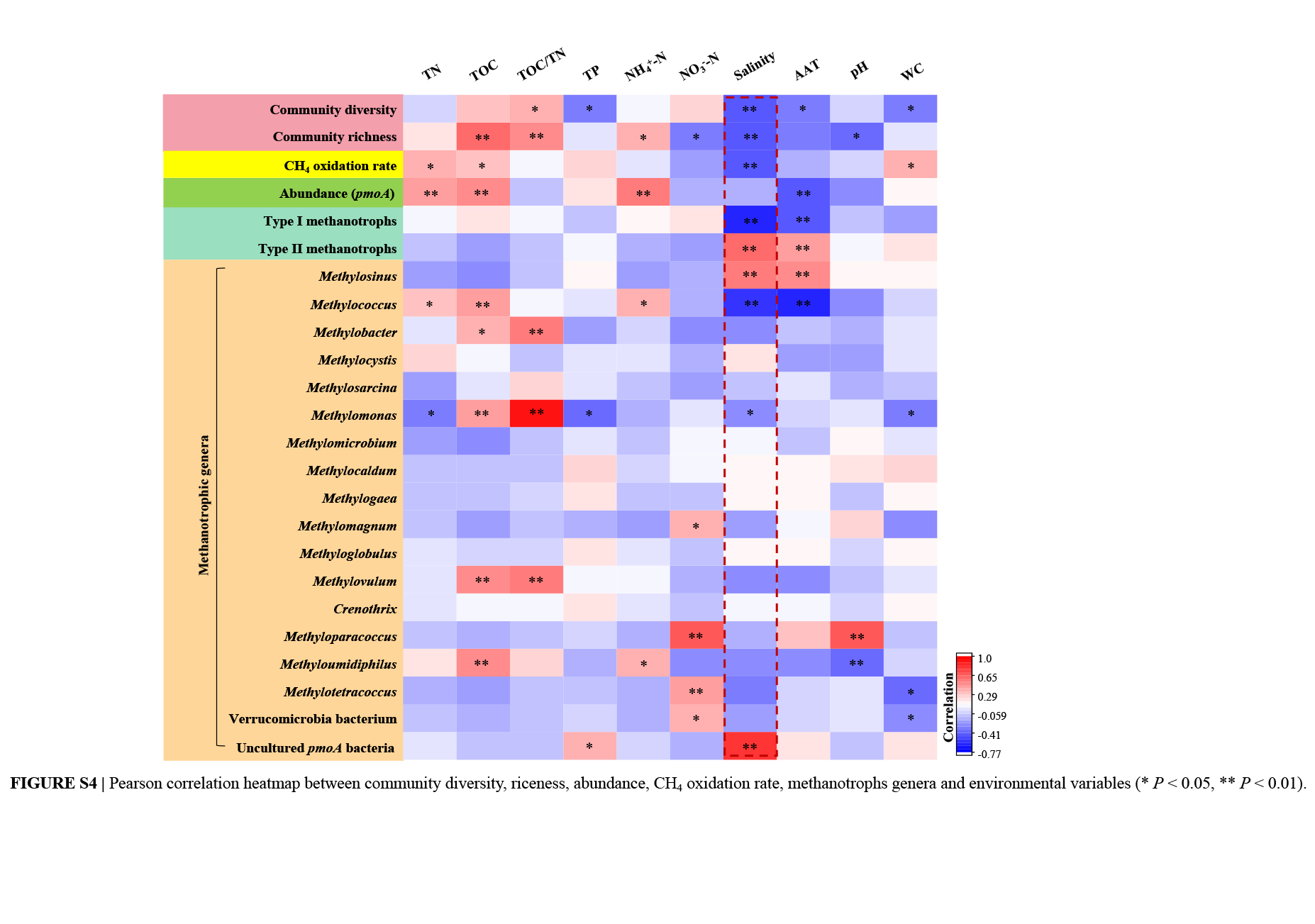

Supplement: Supplementary file 5 [file Image_4.JPEG]

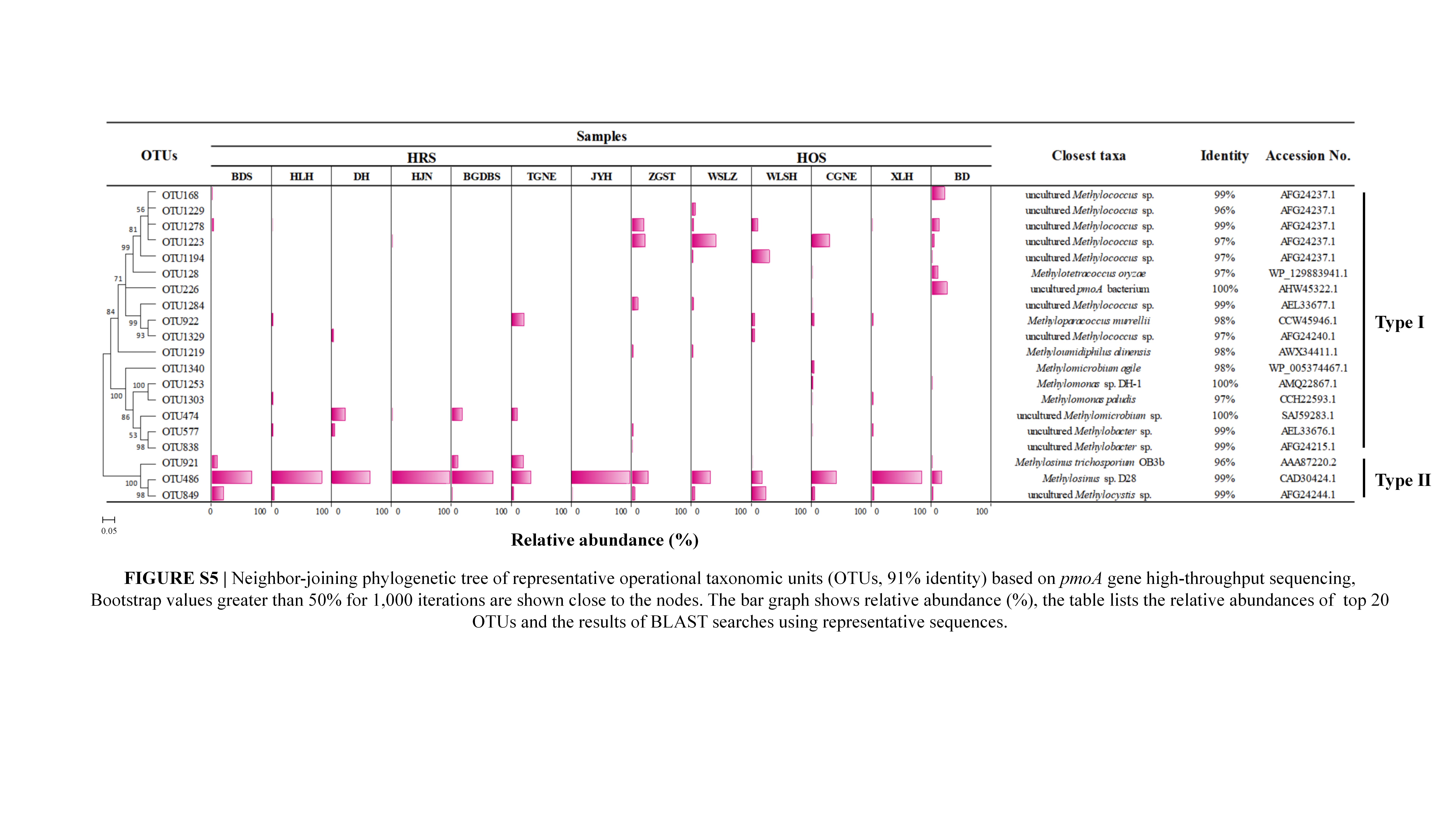

Supplement: Supplementary file 6 [file Image_5.JPEG]

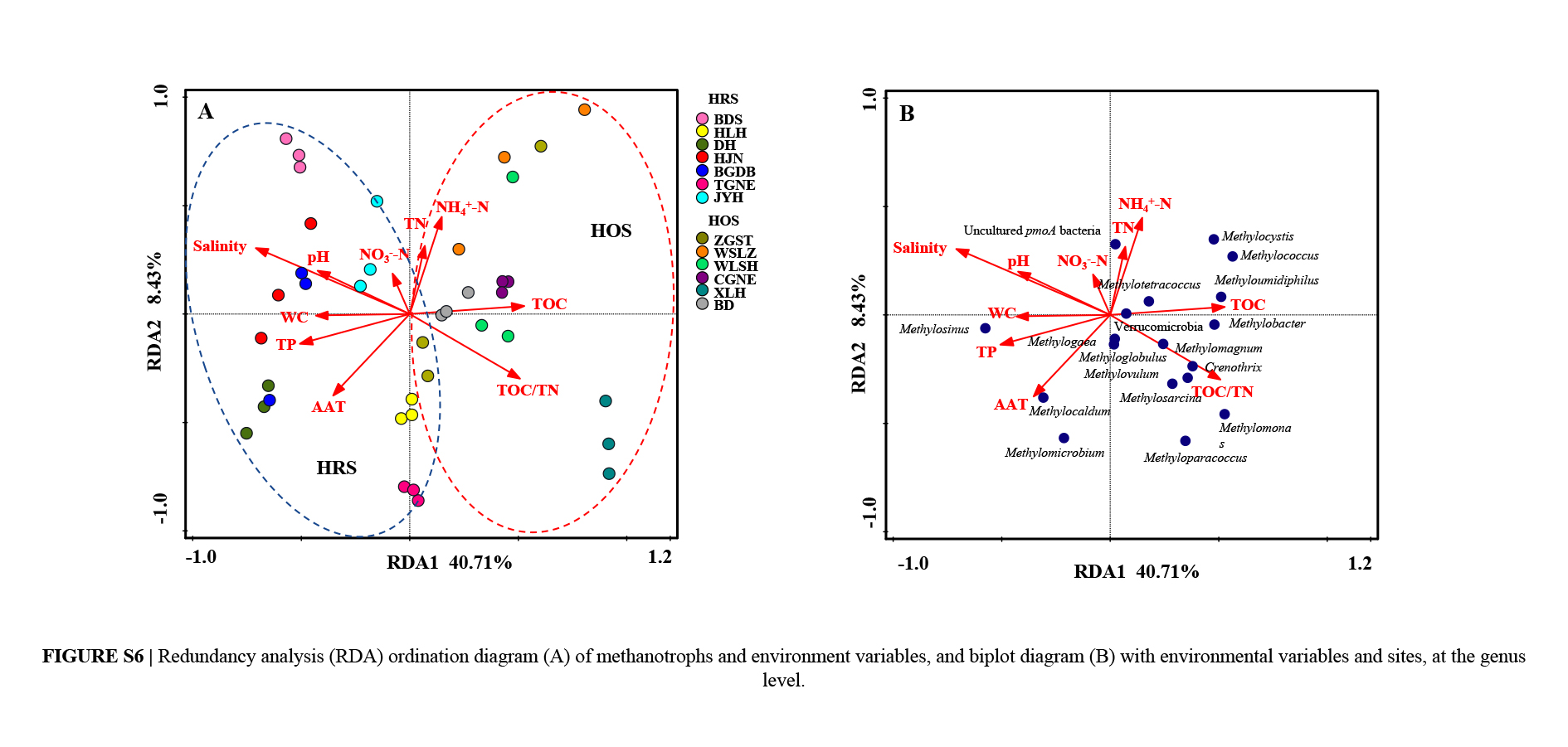

Supplement: Supplementary file 7 [file Image_6.JPEG]
